# Multi-Foci Division of Nonlinear Energy Absorption on Ultrashort Pulse Laser Singulation of Sapphire Wafers

**DOI:** 10.3390/mi12111328

**Published:** 2021-10-28

**Authors:** Celescia Siew Mun Lye, Zhongke Wang, Yee Cheong Lam

**Affiliations:** 1 SIMTech-NTU Joint Laboratory (Precision Machining), Nanyang Technological University, 50 Nanyang Avenue, Singapore 639798, Singapore; celescia001@e.ntu.edu.sg; 2 School of Mechanical and Aerospace Engineering, Nanyang Technological University, 50 Nanyang Avenue, Singapore 639798, Singapore; 3 Singapore Institute of Manufacturing Technology (SIMTech), A*STAR, 2 Fusionopolis Way, Singapore 138634, Singapore

**Keywords:** pulse energy division, spatial proximity of foci, sapphire wafer singulation, surface morphology, multi-foci laser micro processing

## Abstract

The multi-foci division of through thickness nonlinear pulse energy absorption on ultrashort pulse laser singulation of single side polished sapphire wafers has been investigated. Firstly, it disclosed the enhancement of energy absorption by the total internal reflection of the laser beam exiting from an unpolished rough surface. Secondly, by optimizing energy distribution between foci and their proximity, favorable multi-foci energy absorption was induced. Lastly, for effective nonlinear energy absorption for wafer separation, it highlighted the importance of high laser pulse energy fluence at low pulse repetition rates with optimized energy distribution, and the inadequacy of increasing energy deposition through reducing scanning speed alone. This study concluded that for effective wafer separation, despite the lower pulse energy per focus, energy should be divided over more foci with closer spatial proximity. Once the power density per pulse per focus reached a threshold in the order of 1012 W/cm^2^, with approximately 15 μm between two adjacent foci, wafer could be separated with foci evenly distributed over the entire wafer thickness. When the foci spacing reduced to 5 μm, wafer separation could be achieved with pulse energy concentrated only at foci distributed over only the upper or middle one-third wafer thickness.

## 1. Introduction

Sapphire is the key substrate in the manufacturing of light emitting diodes (LEDs), since it is the most economically viable material. Other substrates, such as silicon and silicon carbide, are not suitable for large-scale production of LEDs due to cost and manufacturing quality concerns [1]. Lasers are promising tools for the dicing of brittle sapphire wafers as laser processing reduces cracking and chipping of the products [2,3]. Stealth dicing of transparent materials such as glass and sapphire has been extensively reported as the dicing method can minimize the kerf width and production of top surface debris by focusing the laser within the material thickness [4].

Zhang et al. investigated multiple laser stealth dicing (multi-LSD) with multiple scans in a single scanning line, but with the laser focused within the thickness at different scanning heights [5]. Chang et al. experimented with shifted laser stealth dicing (shifted-LSD) whereby a similar method was employed but shifting the focal position for adjacent scans on different scanning heights [6]. Through these experiments, it has been concluded that a roughened sidewall (i.e., the cleaved surface of the sample) will increase the light extraction of LEDs grown on sapphire substrates. The roughened sidewall reduces the effect of total internal reflection within the LED, allowing more photons to be transmitted out, and therefore improving the light extraction efficiency. However, a single laser focal point within a sapphire wafer would induce uneven thermal and mechanical stress distributions along the wafer thickness direction. As a result, cracks may materialize within a wafer with an unevenly cut sidewall, which is undesirable. Through multifocal optics, the laser energy can be evenly distributed within the material at the various focal points simultaneously. The temperature gradient between the laser focal volume and the surrounding material would be reduced and thus crack formation and deviation can be minimized. As such, multi-foci technology has the potential to produce a controlled roughened sidewall.

Successful multi-foci stealth dicing of transparent glasses and crystals has been reported [7,8]. Ma et al. investigated the dicing of sapphire wafers with a 10 ps laser with 21 foci evenly spaced near the upper section of the sample [9]. However, hitherto only limited investigation on multiple foci dicing of sapphire wafer has been carried out. The effect of the energy level at the multi-foci and spacing between two adjacent foci, which should play dominant roles for successful separation of a wafer, have not been comprehensively investigated. Furthermore, an ultrashort pulse laser of high pulse energy fluence is required to induce nonlinear interaction within an optical transparent material for internal modifications, and thus its micro-processing such as singulation. A lack of understanding of the interaction of these factors becomes an impediment for the optimization of the various laser parameters for a controlled singulation of sapphire. Proper understanding and optimization could result in effective and efficient wafer separation by just concentrating the laser pulse energy to irradiate a section of the thickness instead of distributing the energy evenly over the entire thickness of the wafer.

It has been noted that surface finish in laser machining can be improved by introducing vibration to reduce the debris adhesion to workpiece. Kang et al. explored ultrasonic vibration of the workpiece along the optical axis of the 1070 nm laser beam with a pulse width of 200 ns; spot sizes of 10 μm and 25 μm were employed [10]. Hashemzadeh et al. investigated vibration of the polymethyl methacrylate workpiece parallel to the direction of laser scanning, employing a 70 W continuous wave CO_2_ laser with a 0.2 mm spot size [11]. Both reports indicate improvements in the reduction in debris agglomeration and adhesion onto the work surface, reduction in surface oxidation [10] and increased cutting depth [11] over conventional laser machining without vibration assistance. However, when material separation relies on cleavage by laser internal scribing instead of material removal by laser ablation, vibration may play a different role. Furthermore, additional setup and equipment will be required to introduce vibration to the workpiece, and thus there will be additional complications. Considering the objective of the study reported here, it will be more desirable that the singulation of a sapphire wafer could be achieved without this additional vibration complication.

By employing an ultrashort pulse picosecond laser, this investigation has explored the effect of optimizing the distribution of laser pulse energy over the thickness of the sapphire wafer without the need of sample vibration. Division of pulse energy along the thickness direction was achieved through multi-foci technology; the number of foci and the separation distance between adjacent foci (thus interaction between foci) were dictated by the associated optics combination employed. The effects of pulse energy division over the wafer thickness, the role and importance of the amount of laser energy allocated to each focus and its focus spot size, the distance between two adjacent foci, and the locations of these foci relative to the wafer thickness for achieving wafer cleavage have been studied in detail. By dividing the laser pulse energy over more foci, each with a smaller laser spot size, it could potentially reduce the amount of undesirable damage due to energy absorption over a smaller volume of material (i.e., only at the vicinity of the smaller foci). In addition, our recent study has found that with a rough exit sample surface relative to the laser beam, a larger amount of high intensity laser beam could be totally internally reflected at the exit surface [12]. This effectively traps the laser energy within the sample and reduces the amount of light transmission through the sample, resulting in enhanced nonlinear absorption within the samples that is required for laser machining. To confirm the role of the surface roughness in wafer separation, a sapphire sample with a rough laser beam exiting surface for multi-foci laser processing was compared to a double-side polished sample.

Indeed, by exploiting total internal reflection at the unpolished rough exit surface, and by optimizing the laser beam energy beam distribution, we propose here an innovative approach for efficient and effective wafer singulation by dividing and concentrating the pulse energy to only the middle one-third section of the wafer thickness over many foci with close spatial proximity. In this investigation, pulse energy division refers to the energy of a single pulse divided over many foci along the wafer thickness, and spatial proximity of foci refers to the distance between two adjacent foci.

## 2. Materials and Methods

The 0.43 mm thick single crystal ${\mathrm{Al}}_{2}O_{3}$ sapphire wafers from Latech Scientific Supply Pte. Ltd. (Singapore) were employed. The sapphire samples had a c-plane crystal orientation and refractive index of 1.768. The single-side polished sapphire samples were oriented such that their roughened surface faced away from the laser. For comparison, a double-side polished sapphire sample was used as a reference. The measured roughness of each surface was an average of 12 measurements, i.e., 6 measurements each in the 0° and 90° directions, respectively. The average surface roughness Ra and Rz for a polished surface were 1.93 nm (standard deviation 0.00015 μm) and 9.80 nm (standard deviation 0.00061 μm) respectively; the average surface roughness Ra and Rz for an unpolished surface were 0.954 μm (standard deviation 0.059 μm) and 6.74 μm (standard deviation 0.544 μm) respectively.

**Figure 1:**
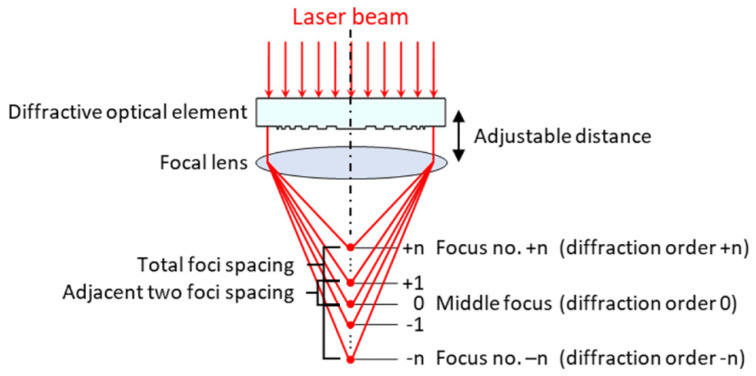
Schematic illustration of generation of multiple foci with a combination of optical diffractive element and focal lens.

A 10.3 ps Nd:YAG laser from Time-Bandwidth Products AG (Zurich, Switzerland) was employed to scan the sapphire wafer. The laser had a 1064 nm wavelength, a 6.5 mm unfocused beam diameter with a M^2^ value of 1.3. The multiple foci were obtained through multi-focal optics (multi-focus lens, MF) consisting of a combination of a diffractive optical element (DOE) and a conventional focusing lens, as shown in Figure 1.

The distance between the DOE and the focusing lens can be arbitrary and is not a key parameter as the phase of the laser beam only changes after propagating through the diffractive elements [13]. The effective focal length (EFL) of the focusing lens dictates the average spacing between two adjacent foci (i.e., the foci spacing); the total foci spacing is defined as the distance between the focus closest to the laser source to the focus furthest from the laser source. A shorter EFL resulted in a shorter foci spacing, and a longer EFL a longer foci spacing. The focus of diffraction order 0 (i.e., the middle focus) is located along the optical axis at the EFL. Foci closer to the laser source have positive diffraction orders and foci further from the laser source have negative diffraction orders, with diffraction orders numerically increasing away from diffractive order 0.

To investigate the pulse energy distribution over the sapphire wafer thickness, the influence of foci number which determines the magnitude of pulse energy per foci (estimated by pulse energy over foci number), the foci spot diameter and the spacing between two adjacent foci which determine the spatial proximity of energy division have been studied experimentally. Two types of diffractive optical elements for 27 foci and 9 foci, respectively, from HOLO/OR Ltd., Israel, were employed; 27 foci would divide the energy over more foci resulting in lower energy per focus, and the converse is true for 9 foci. Three types of focal length lenses were employed with regards to the number of foci and their separation distance. Two conventional focal lenses with EFL 12 and 7.5 mm from Edmund Optics, Singapore, were selected to produce a total foci spacing close to the entire thickness of the sapphire wafer, namely the combinations of DOE 27 foci with EFL 7.5 mm, and DOE 9 foci with EFL 12 mm, respectively. This will result in two different adjacent foci spacing in the through thickness direction, with larger adjacent foci spacing for DOE 9 foci (thus larger distance between the pulse energy concentrated at the foci), and shorter adjacent foci spacing for DOE 27 foci (thus a smaller distance between the pulse energy concentrated at the foci). To further reduce the adjacent foci spacing of DOE 27, an OB40× objective lens from Meiji Techno, USA, was employed as it had a much shorter EFL which further reduced the distance between two adjacent foci. In addition, the different optical setups would result in different foci spot size. The details of the optics combination with the associated foci diameter and laser processing parameters are shown in Table 1.

**Figure 2:**
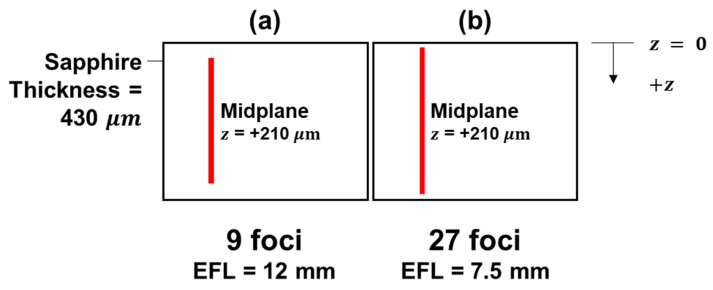
Schematic of scanning over the full thickness with diffractive order 0 positioned at z value = +210 μ m for (**a**) 9-foci setup (Experiment F9) and (**b**) 27-foci setup (Experiment F27), respectively.

**Figure 3:**
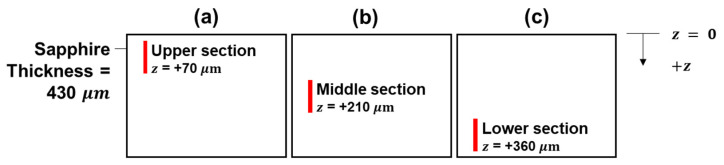
Schematic of the three scanning sections over one-third thickness of the sample with diffractive order 0 positioned at the z values for (**a**) lower (Experiment L27), (**b**) middle (Experiment M27) and (**c**) upper (Experiment U27) sections, respectively.

In the experiments, all foci were placed within the interior of the wafer thickness under a single pass laser beam scanning at different speeds of 0.1, 1 and 10 mm/s. Figure 2 and Figure 3 show the schematics of the scanning sections for full-section scanning (i.e., Experiments F9 and F27) and partial one-third section scanning experiments (i.e., Experiments L27, M27 and U27) respectively, with the vertical red line signifying the locations of the foci in each experiment. The respective z value (i.e., the position of diffraction order 0) with respect to the top surface of the sample (with the positive direction into the sample) indicates the location of the scanning section within the thickness of the sapphire wafer.

## 3. Results and Discussion

### 3.1. Pulse Energy Nonlinear Absorption Enhancement

The study on pulse energy nonlinear absorption enhancement was carried out through the investigation of the effect of wafer surface roughness by the comparison of single and double-side polished sapphire wafer samples.

**Figure 4:**
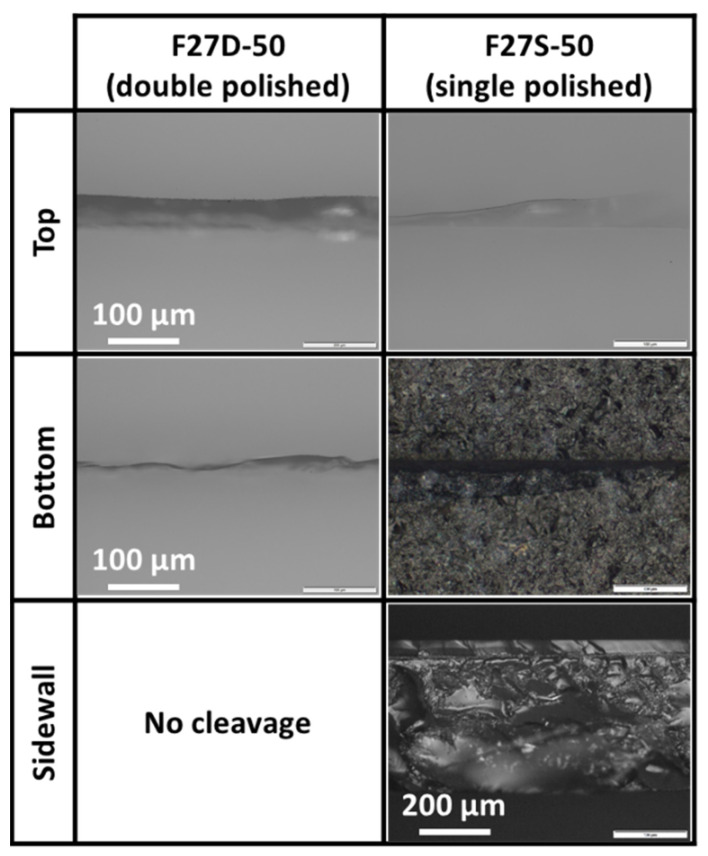
Optical images of samples’ surfaces and sidewall profiles at 0.1 mm/s scanning speed for Experiments F27D-50 and F27S-50 (i.e., after full-section irradiation with 27 foci and a 7.5 mm EFL at a pulse repetition rate of 50 kHz at 1.13 W).

The scanning of a double-side polished sample was first investigated. Experiment F27D-50 was carried out on a double-side polished sample at 0.1 mm/s scanning speed at 1.13 W. A low repetition rate of 50 kHz was chosen to produce the highest peak power possible; the slowest scanning speed of 0.1 mm/s was chosen for its high energy deposition into the sample, thus giving the best opportunity for nonlinear modification to occur during laser irradiation. In accordance with our recent investigation on sample surface roughness [12], it is to be expected that without the rough exit surface, only cracks were observed on both surfaces of the sample after laser irradiation as shown in Figure 4. Mechanical cleavage was unsuccessful for all samples due to insufficient weakening of the samples.

The lack of ablation scribes at the bottom rough surface was attributed to the lack of sufficient energy to induce nonlinear absorption within the sample. Without the bottom rough surface, total internal reflection at the exit surface was impossible. Although reflection of light at an interface occurred naturally, the amount of accumulated energy from the reflected laser beam at the bottom surface and the incoming laser beam was still insufficient to induce ablation on the bottom surface. As such, the energy deposited only resulted in the surface cracks observed.

To evaluate the effect of a rough exit surface, the scanning of a single-side polished sample was investigated. Experiment F27S-50 was carried with the same laser experimental parameters as that of Experiment F27D-50, except that the sample was a single-side polished sample. By simply having a bottom unpolished exit surface in Experiments F27S-50, there was total internal reflection of the laser beam at the exit surface. As discussed in our recent investigation [12], the rough exit surface caused the laser beam to form different angles of incidence at the exit surface. When the angle of incidence of a laser beam exceeds the critical angle, total internal reflection occurs. The laser energy is effectively trapped within the medium with the reflection of the light beam back into the sample. With sufficient energy accumulating, nonlinear absorption could occur. Lye et al. reported a 40% decrease in transmitted power once the nonlinear absorption threshold has been surpassed, indicating the existence of nonlinear absorption within the sample for laser power exceeding this threshold [12]. With an increase in laser power, more energy is accumulated increasingly at the vicinity of the rough exit bottom surface where total internal reflection occurred. This enables the formation of the ablation-dominated scribes at the exit bottom surface in addition to that at the top polish surface as shown in Figure 4. Furthermore, the accumulation of energy due to total internal reflection sufficiently weakened the samples and sample separation was achieved. These experiments demonstrated the benefits of employing a sample with a rough unpolished exit surface for laser processing.

As both (single- and double-side polished) samples had a top polished surface, the condition at the top surface and the interior of the sample would be similar for both F27S-50 and F27D-50 experiments. Since the only difference lies in the surface finish on the bottom surface, and thus the possibility of total internal reflection associated with light exiting a rough unpolished surface, any divergence in the results between F27S-50 and F27D-50 could be attributed to the rough exit surface. In addition to saving in polishing costs by using a single-side polished sample as compared to a double-side polished sample, the different outcomes between these two experiments vividly demonstrate the benefit of having a rough exit surface for laser processing.

With the benefit of an unpolished exit surface established, the nonlinear absorption of the pulse energy could be enhanced to assist laser processing through the total internal reflection of a laser beam exiting from an unpolished rough surface. Thus, all subsequent experiments were carried out on a single-side polish sample with a rough exit surface.

### 3.2. Pulse Energy Division along Wafer Thickness

For sapphire wafer cleavage, it seems to be an advantage to have a uniform thickness distribution of the laser pulse energy over the entire wafer thickness. This could be achieved by dividing the pulse energy over as many foci as possible in the through thickness direction. In this investigation, this was achieved by splitting the single pulse energy and distributing it over 27 foci. By selecting the focal lens with the appropriate 7.5 mm EFL resulting in close spatial proximity between adjacent foci of approximately 15 μm (as shown in Table 1), all 27 foci could be focused within the wafer thickness of 430 μm.

**Figure 5:**
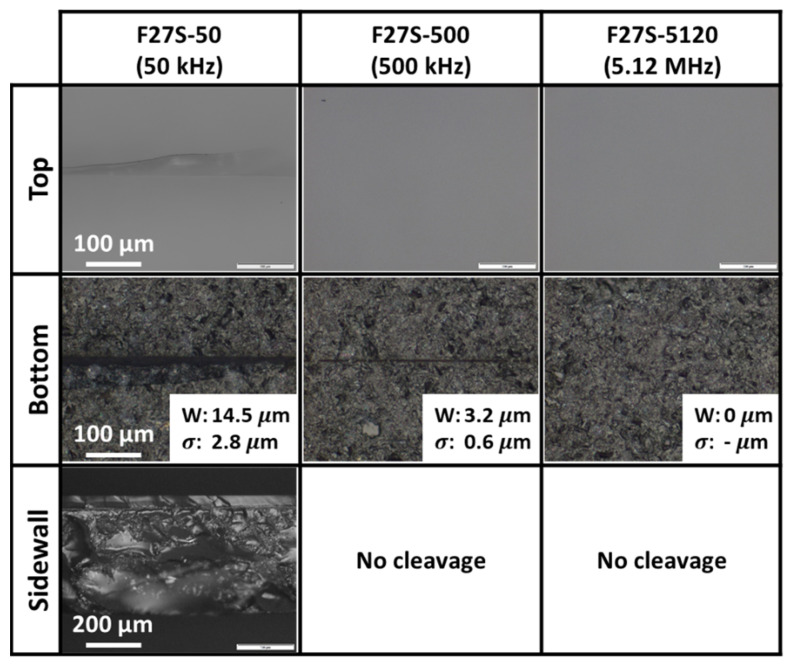
Optical images of samples’ surfaces and sidewall profiles at 0.1 mm/s scanning speed for Experiment F27S (i.e., after full-section irradiation with 27 foci and a 7.5 mm EFL at 1.13 W) at various pulse repetition rates (i.e., 50, 500 and 5120 kHz), with the respective widths (W) and standard deviations (σ).

Figure 5 shows the optical images of the samples’ surfaces and sidewall profiles of the cleaved samples after laser irradiation at 0.1 mm/s scanning speed and various pulse repetition rates for Experiment F27S (i.e., after full-section irradiation with 27 foci and a 7.5 mm EFL at 1.13 W). The average widths and their standard deviations of the surface scribes (if any) are indicated in the figure. Each average and standard deviation was calculated from three measurements of a scribe width. Single-side polished samples with a rough exit surface were employed.

Figure 5 shows that no scribes could be observed on the top polished entry surface regardless of pulse repetition rate, indicating insufficient pulse energy deposited at the top surface. In contrast, ablation scribes were observed at the bottom rough unpolished exit surface for lower repetition rates of 50 and 500 kHz in Experiments F27S-50 and F27S-500, respectively. The presence of the rough exit surface enhanced the occurrence of total internal reflection. The interaction between the totally internally reflected laser beam and the incoming laser beam accumulated the laser energy of high intensity in the region at the rough exit surface. Ablation scribes were only formed when the amount of high intensity accumulated energy exceeded the threshold for ablation scribe formation. Increasing the pulse repetition rate reduced the pulse energy, decreasing the amount of high intensity laser energy accumulated, and thinner ablation scribes were formed. Furthermore, as a result of the higher pulse energy deposition within the sapphire due to total internal reflection, a crack was observed on the top surface at high pulse energy fluence for Experiment F27S-50 (i.e., 594.83 J/cm^2^ at 50 kHz repetition rate in Table 1).

By keeping the laser power output constant, further increasing the pulse repetition rate to 5.12 MHz in Experiment F27S-5120 reduced the energy of each pulse (5.809 J/cm^2^ at 5.12 MHz). Even with the total internal reflection of the laser beam at the rough exit surface, the laser energy accumulated was of low intensity and could not induce the formation of an ablation scribe.

It was further revealed that only the sample at high pulse energy (594.83 J/cm^2^ at 50 kHz) in Experiment F27S-50 could be mechanically cleaved as shown in Figure 5. The low pulse repetition rate produced pulses with higher pulse energy for nonlinear modification of the sample. The nonlinear modification sufficiently weakened the sample such that mechanical cleavage could be successfully performed on the sample.

Although bottom surface scribes were observed for Experiment F27S-500, the low pulse energy could not induce significant nonlinear modification to sufficiently weaken the sample for mechanical cleavage. This demonstrates that the observation of an ablation scribe was not a sufficient indication that a sample could be successfully cleaved.

These experiments indicate that uniform fine pulse energy division over the sapphire wafer thickness would be necessary, and a high pulse energy at a low pulse repetition rate was essential for sufficient nonlinear modification of the sample required for sapphire wafer separation. As such, subsequent experiments would only be conducted at a 50 kHz pulse repetition rate.

### 3.3. Optimal Spatial Division of Pulse Energy along Wafer Thickness

The optimal level of pulse energy spatial division over the sapphire wafer thickness was investigated through a study of the number of foci and foci spacing in multi-foci full-section scanning. By employing MF lenses of 9 foci and 27 foci (three times of 9 foci), rather different spatial distributions and uniformities of laser energy across the entire wafer thickness were realized for Experiments F9S-50 (9 foci) and F27S-50 (27 foci), respectively; see Table 1. Single-side polished samples with a rough exit surface were employed as suggested in Section 3.1. The laser power employed was 1.13 W at a 50 kHz pulse repetition rate, as it was determined to be critical to have a high peak intensity to induce nonlinear absorption [12].

The laser produced 22.6 μJ for each pulse and the amount of pulse energy allocated to a single focus was dependent on the number of foci formed by the MF lens. Each focus formed by the 9-foci lens had approximately triple the energy amount of a single focus formed by the 27-foci lens, with pulse energies of 2.51 μJ and 0.837 μJ, respectively.

However, since focusing optics of different EFL were employed for the respective MF lenses, as shown in Table 1, the focused spot diameter for the 9-foci lens was larger than the 27 foci lens, i.e., 3.52 μm for the 9 foci lens and 2.20 μm for the 27-foci lens. The spot size has a direct impact on power density. Accounting for the difference in the laser power per pulse per focus and the focused spot size, and despite the larger focus spot size, as shown in Table 1, each focus in Experiment F9S-50 has a higher power density per pulse per focus of 2.51 TW/cm^2^ as compared to 2.14 TW/cm^2^ of Experiment F27S-50. Although the difference in power density per pulse per focus was only approximately 17%, this could result in a significant difference in nonlinear absorption behavior as nonlinear multiphoton absorption is expected to be proportional to the laser intensity to the power of number of photons simultaneously absorbed [14,15]:

(1) $$\begin{array}{c} dI/dz=-\alpha I^{n} \end{array}$$

where I is the power density, z the distance along the optical axis, α the nonlinear absorption coefficient and n the number of photons simultaneously absorbed in multiphoton absorption. For the current investigation, the irradiation of sapphire under a 1064 nm laser resulted in a nine-photon absorption (9PA) process (i.e., n = 9) [15].

The intensity at a focus spot is proportional to the laser power, and inversely proportional to the number of foci and the focus spot area. As such, for the same laser power applied, the ratio of intensity *I* and dI/dz for a single focus in Experiment F9S-50 (with 9 foci and a focus spot diameter of 3.52 μm) to that of F27S-50 (with 27 foci and a focus spot diameter of 2.20 μm) may be calculated to be approximately 1.17 and 4.2 times, respectively. This implies that a significant amount of energy would be absorbed over a much shorter distance for F9S-50 as compared to F27S-50. This higher energy absorption over a shorter distance would result in higher heating and thus thermal stress for F9S-50. In addition, the larger focus spot diameter of F9S-50 compared to F27S-50 would also mean that a larger area in the x–y plane parallel to the surface of the sample would also experience the higher heating and thermal stress for F9S-50.

On closer examination, the distribution of the energy absorbed was rather different for these two experimental setups. With a large foci spot diameter and area, F9S-50 would have a larger proportion of the absorbed energy distributed in the x–y plane parallel to the surface of the sample than that of F27S-50. In contrast, with more foci, F27S-50 would have a higher proportion of absorbed energy which is more evenly distributed in the through thickness direction of the sample compared to F9S-50; intuitively, one would expect that this favorable concentration and spatial division of pulse energy of F27S-50 along the through thickness covering the entire wafer thickness would promote wafer singulation as wafer cleavage is a “through thickness” operation.

As such, this study further identified the effects of the spatial proximity of focused pulse energy division through (a) larger spatial distance between two adjacent foci by splitting laser pulse energy over fewer foci, each with higher power density (overall more energy absorbed per focus), i.e., Experiment F9S-50 or (b) closer spatial distance between two adjacent foci by splitting laser pulse energy over more foci, each with lower intensity and energy absorbed for each focus but with a higher proportion of the absorbed energy more evenly distributed along the desirable through thickness direction, i.e., Experiment F27S-50.

The effects of energy deposition within the samples on scribe formation were further investigated by changing the laser scanning speeds (i.e., 0.1, 1 and 10 mm/s). The amount of energy deposited can be derived as [16,17]:

(2) $$\begin{array}{c} E_{d}=QN\\ \;\;\;\;=\frac{P}{R_{p}}\times \frac{R_{p}\phi }{v} \end{array}$$

(3) $$\begin{array}{c} E_{d}=\frac{P\phi }{v} \end{array}$$

where $E_{d}$ is the energy deposited within the sample, Q the pulse energy, N the number of consecutive laser pulses that overlap in a single spot size, P the average power, $R_{p}$ the pulse repetition rate, ϕ the focused spot diameter and v the laser scanning speed. For the same laser power and focused spot diameter, Equation (3) indicated that the energy deposited into the sample decreases with scanning speed, and it does not depend on the repetition rate.

**Figure 6:**
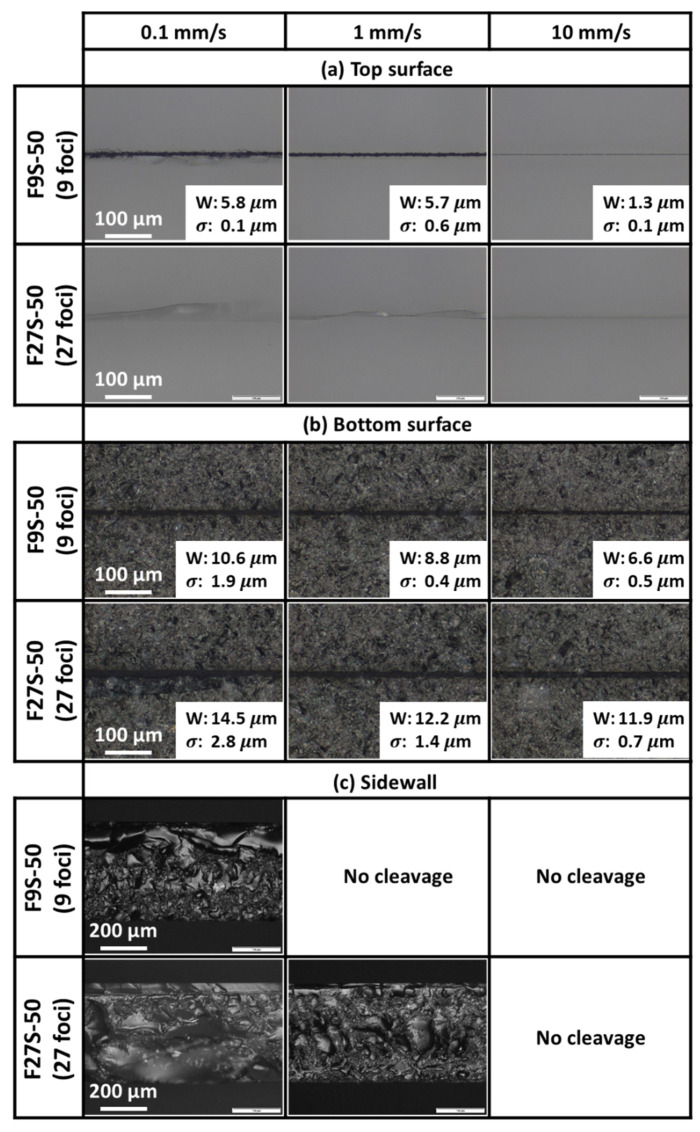
Optical images of (**a**) top polished entry surface, (**b**) bottom unpolished exit surface and (**c**) sidewall profiles after full-section scanning at 1.13 W with the respective widths (W) and standard deviations (σ).

Figure 6a shows the optical images of the top polished entry surface after the laser irradiation at various scanning speeds for the full-section scanning experiments. The widths and their standard deviations of the top surface scribes (if any) are indicated in the figure.

As shown in Figure 6a, samples of Experiment F9S-50 had scribes on the top polished entry surface for all scanning speeds. This is somewhat unexpected as the total foci spacing only occupied approximately 80% of the thickness of the sample, with no foci in close proximity to the surfaces. Indeed, this illustrated the importance of having sufficient laser power for scribe formation even when the laser foci are not near the surface.

Crack-dominated scribes were formed at the slower scanning speeds of 0.1 and 1 mm/s, while ablation scribes were formed at the faster scanning speed of 10 mm/s. When the scanning speed increased from 0.1 to 10 mm/s, less energy was deposited into the samples and the scribe widths decreased. The different types of scribe formation were dependent on the amount of energy deposition.

At the slower scanning speeds (i.e., 0.1 and 1 mm/s), the energy deposited internally modified the sample and the excess energy was channeled to the formation and propagation of internal cracks. Since crack propagation could not be controlled, deviated cracks were formed on the top surface. The internal cracks acted as micro-surfaces that reflected the incoming laser beam onto other micro-surfaces, effectively trapping light within the region. Additionally, since the laser energy was split only between 9 foci, the energy allocated to a single focus was higher (as compared to 27 foci). The combination of the two effects accumulated energy near the top surface by the unfocused laser. With sufficient energy accumulated, ablation occurred that resulted in a crack-dominated scribe forming on the top surface.

At the 10 mm/s scanning speed, the energy deposited was just sufficient for the formation of a thin ablation-dominated scribe on the top polished surface of the sample. The amount of energy deposited was not in excess to form cracks seen in the samples irradiated with slower scanning speeds.

In contrast, as shown in Figure 6a, in Experiment F27S-50, the total foci spacing occupied approximately 90% of the sample thickness and had close proximity between two adjacent foci. Although some foci were close to the top polished entry surface, no scribes were observed. This could be attributed to the lower energy allocated to a single focus which could not induce scribe formation. Instead, cracks were observed on the top surface with the occasional subsurface damage for all scanning speeds. As the scanning speed was increased, less excess energy was deposited into the samples to form and propagate the internal cracks to the top surface. The amount of excess energy deposited to the samples dictated the length of cracks formed and the extent of crack deviation from the scanning direction.

Figure 6b shows the optical images of the bottom unpolished exit surface after laser irradiation at various scanning speeds for full-section scanning experiments. The widths and their standard deviations of the bottom surface scribes (if any) are indicated in the figure.

As shown in Figure 6b, ablation-dominated scribes were formed on the bottom unpolished rough surface for all scanning speeds in both Experiments F9S-50 and F27S-50. Figure 6b indicates that the bottom-surface scribes were always thicker than the top-surface scribes shown in Figure 6a regardless of the scanning speed employed. Even though the spot size of each foci remained relatively constant, the topology of the roughened surface affected the overall effective area irradiated by the laser beam—the peaks and troughs of a roughened surface increased the irradiated area. With total internal reflection at the exit surface, some of the reflected laser beam could have been further reflected off the adjacent surface, depending on the surrounding topology, thus accumulating energy in this region. With the laser beam diffused over a larger area with larger energy accumulation caused by total internal reflection, a thicker scribe could be formed on the bottom surface.

With increasing scanning speed, both Experiment F9S-50 and Experiment F27S-50 produced scribes with decreasing widths. As the energy deposited decreases with scanning speed, this indicates that the width of the scribes was a function of energy deposition.

The greater the distance the laser beam of the last focus had to travel to the bottom surface, the greater the energy loss through linear absorption. As such, it is expected that the width of the scribe should also be a function of the proximity of the foci to the surface and their allocated energy. The last focus of the 9-foci setup in Experiment F9S was further from the bottom surface than the last focus of the 27-foci setup in Experiment F27S. Indeed, consistently, at the same scanning speed, the widths of the ablation scribes of Experiment F9S-50 were narrower than that of F27S-50.

It should also be noted that only at the slowest 0.1 mm/s scanning speed in Experiment F27S-50 was there sufficient excess energy to propagate the internal cracks to the bottom surface. Cracks that largely deviated from the scanning direction were observed adjacent to the scribe.

Figure 6c shows the optical images of the sidewall profiles of the successfully cleaved samples after full-section scanning with different scanning speeds. The sample irradiated at the 0.1 mm/s scanning speed in Experiment F9S-50 was mechanically cleaved. The sidewall profile revealed large wave-like structures in the region closest to the top surface of the sample, justifying the cracks observed on the top surface. The optical image can be observed to have two types of structures near the bottom rough surface of the sample, namely larger wave-like cracks formed by mechanical cleavage and smaller wave-like cracks formed by laser modification of the material. Closer inspection revealed that the smaller wave-like structures formed by laser modification of the material were not on the cleavage plane. This further confirmed that the excess energy deposited on the material resulted in the formation of internal cracks. The formation of the two distinct regions was dictated by the amount of energy each focus had at its focus position. The closer the focus was to the top surface, the shorter the distance it had to travel and the amount of energy lost through linear absorption by the sample was reduced. Therefore, there was more excess energy to form both laser-modified areas and larger internal cracks. The converse was true; focus further from the top surface of the sample would have less excess energy to form large internal cracks after modification.

As shown in Figure 6c, samples irradiated at a scanning speed of either 0.1 or 1 mm/s in Experiment F27S-50 with more uniform concentrated pulse energy distribution over a larger number of foci were successfully cleaved. At the 0.1 mm/s scanning speed, the sidewall profile shows that the plane of cleavage was not perfectly perpendicular to the top and bottom surfaces of the sample. A plausible explanation could be the randomness in internal cracks formation from the excess energy deposition to the sample. As such, mechanical cleavage would tend to propagate along the weakened paths of the internal cracks instead of along the plane of laser modification. Since the internal cracks were formed in a random manner, the plane of cleavage was not perfectly perpendicular to the surfaces of the sample.

At the 1 mm/s scanning speed with less excess energy deposited, the optical image of the sidewall profile displayed a plane of cleavage that seemed to be along, or closer to, the plane of laser modification. The nonuniformly roughened sidewall profile seemed to contain smaller cracks in the plane of laser modification. Therefore, the sidewall profile observed could contain both the laser modified areas and small internal cracks.

For samples irradiated by a 1 mm/s scanning speed, the 9-foci setup in Experiment F9S-50 provided a larger amount of energy deposited to the samples than the 27-foci setup in Experiment F27S-50. Yet mechanical cleavage of the samples was only successful for the Experiment F27S-50 samples but not for the Experiment F9S-50 samples. This illustrates the importance of the role of energy distribution and foci spacing. It was more favorable to split the laser energy more uniformly over more foci with closer proximity between adjacent foci along the desirable through thickness direction as in Experiment F27S-50.

Although each focus in Experiment F27S-50 had a lower pulse energy, smaller spot size and less absorption, a larger proportion of the energy was distributed in the through thickness direction with the foci closely spaced to each other and effectively interacted with the adjacent foci compared to Experiment F9S-50. From the sidewall profile of the sample in Experiment F27S-50 with 1 mm/s scanning speed, the clear observation of the small wave-like internal cracks shows that the cleavage plane corresponded to the laser scribing plane, i.e., the plane containing the laser optical axis. As demonstrated, although there was less energy absorption at each focus, the more evenly distributed absorbed energy along the desired direction was more ideal as there was potentially less undesired damage (such as undesired cracking) to the sample. With a closer proximity between foci, the desirable separation cracks in the through thickness direction induced by one focus could extend to other cracks induced by an adjacent focus. Since the 27 foci were more evenly spaced over the thickness of the sample, it effectively formed a line of cracks in the plane of the laser optical axis in the through thickness direction of the sample. These crack interactions sufficiently weakened the sample for mechanical cleavage.

In contrast, in Experiment F9S-50, with a higher pulse energy, larger spot size and more energy absorption over a shorter through thickness distance, it might cause greater undesirable damage to a larger area as a much larger proportion of energy absorbed was distributed along the x–y plane parallel to the sample surface. The larger spacing between foci meant that interaction between the separation cracks induced by the foci was minimal along the optical axis in the through thickness direction of the sample. As such, mechanical cleavage could well not be successfully completed at a higher scanning speed. This conclusion was only valid if each focus had an appropriately high intensity for nonlinear absorption. Hence, employing the 27-foci setup in Experiment F27S was a more effective use of laser energy as sample cleavage was possible with less energy deposition. The unsuccessful cleavage of samples irradiated at a scanning speed of 1 or 10 mm/s in Experiment F9S-50 or 10 mm/s scanning speed in Experiment F27S-50 may be attributed to the insufficient energy deposited at the higher scanning speed; the samples could not be effectively weakened for cleavage.

This study clearly concluded that finer energy division by splitting a laser beam into more foci, with closer proximity between two adjacent foci resulting in a more uniform concentration of pulse energy in the through thickness direction, is highly desirable and effective for the singulation of sapphire wafers even at lower pulse energy per focus. This is provided that the power density per pulse per focus reached the threshold in the order of approximately 10^12^ W/cm^2^.

### 3.4. Cleavage with Pulse Energy Allocation Concentrated over Only One-Third Section of Wafer Thickness

Three major deductions may be drawn from the observations elaborated in the previous sections. First, there is total internal reflection at the unpolished rough exit surface. This could and should be exploited; namely the laser energy may not have to be focused at the bottom (i.e., near the exit surface) region and the reflection should be allowed to amplify the laser energy for this region. Second, as the pulse energy was divided over more foci with a smaller separation between two adjacent foci, there would be effective interaction between foci, resulting in desirable outcomes. A higher proportion of energy absorbed would be more evenly distributed along the desirable thickness direction. This could be achieved much more effectively with the same number of 27 foci if they were distributed only over the partial thickness of the sample instead of the full thickness of the sample, resulting in much closer proximity between foci. This is particularly applicable for a brittle material such as sapphire. Third, the focus spot diameter could be reduced further to increase the pulse energy intensity for a given laser power for efficient nonlinear absorption over a small distance and volume to generate the desirable and concentrated thermal stress distribution along the wafer thickness direction for singulation. Together with proper exploitation of total internal reflection and smaller foci spacing, and without having the pulse energy distributed over the entire thickness, lower input laser power could well minimize the undesirable damage and achieve a better outcome with efficient laser power utilization.

To verify the above deductions, instead of scanning the entire wafer thickness, only a one-third section of the wafer thickness was scanned. Three different experiments were performed, namely upper, middle and lower partial one-third section scanning experiments. To achieve close proximity between foci, 27 foci were employed for pulse energy division along the wafer thickness. A short EFL of 4.38 mm, resulting in approximately 5 μm between two adjacent foci, was employed to compress the 27 foci over the one-third section of the wafer. In addition, even with a 30% reduction in laser power to 0.80 W instead of the 1.13 W used previously, the much smaller focused spot size of just 1.29 μm from EFL of 4.38 mm could still produce a power density per pulse per focus in the order of 10^12^ W/cm^2^, the threshold required for wafer separation.

With the same reasoning as before, dI/dz in the one-third section experiments was more than six hundred times greater compared to the full section experiments; together with a much smaller focus spot size, the absorbed energy would be concentrated over a much smaller volume generating high thermal stress for sapphire wafer cleavage. As the spatial proximity of energy division through foci separation was much closer for the one-third section experiments, it can be expected that this higher thermal stress would be much more evenly distributed over the one-third section along the through thickness direction irradiated by the laser beam.

**Figure 7:**
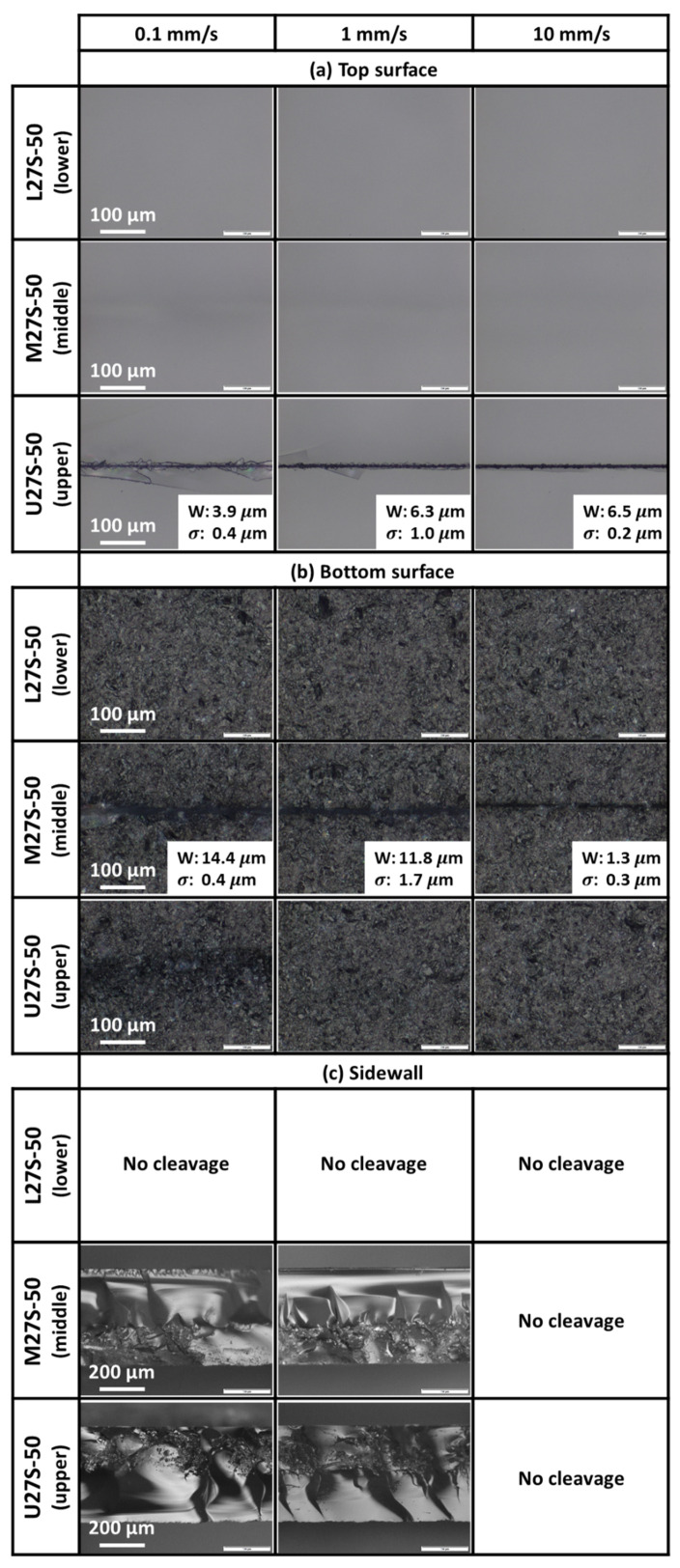
Optical images of (**a**) top polished entry surface, (**b**) bottom unpolished exit surface and (**c**) sidewall profiles after one-third section at 0.80 W scanning with the respective widths (W) and standard deviations (σ).

Figure 7a shows the optical images of the top polished entry surface after laser irradiation at various scanning speeds for one-third section scanning experiments. The widths and their standard deviations of the top surface scribes (if any) are indicated in the figure.

When the pulse energy was concentrated at the lower section in the sapphire wafer thickness direction through placing the foci at the lower section, which was rather far away from the top surface, there were no visible changes observed on the top polished surface. Due to the large distance between the locations where energy was absorbed and the wafer top surface, it was impossible for the pulse energy to interact with the top surface for scribe formation or observable subsurface damages for all scanning speeds.

Similarly, the laser irradiation of the middle section also produced no visible scribes on the top polished surface. Compared to the lower one-third section scanning, the foci were closer to the top surface. This resulted in observable subsurface damages on the top surface for all scanning speeds. The most distinct subsurface damage was observed at the slowest scanning speed of 0.1 mm/s. With a slow scanning speed and thus more energy deposition, there was more internal modification of the sample and thus more distinct subsurface damage could be observed.

Laser irradiation of the upper section resulted in crack-dominated scribe formation due to the close proximity between the absorbed energy location and the top polished surface. As the scanning speed increased, less energy was deposited into the sample and there was less excess energy to form and propagate cracks to the top surface. Therefore, the lengths of cracks and extent of crack deviation from the scanning direction would decrease.

Figure 7b shows the optical images of the bottom unpolished exit surface after the laser irradiation at various scanning speeds for the one-third section scanning experiments. The widths and their standard deviations of the bottom surface scribes (if any) are indicated in the figure.

With the pulse energy absorbed by foci positioned at the lower section, one would expect the formation of ablation scribes at the bottom rough surface. However, visible scribes could not be observed at any scanning speeds. With multi-foci, the pulse energy of each focus was much lower than the total laser energy per pulse. Thus, a plausible explanation was that a significant percentage of the laser energy of each focus was lost through linear absorption by the bulk thickness when the laser beam propagated through the sample. When the laser beam eventually focused at the lower section, even with total internal reflection at the bottom surface, there was insufficient energy accumulated to induce nonlinear absorption at the bottom surface and no scribes were formed.

Interestingly, when the foci were focused at the middle section, which was far from the bottom rough surface, some ablation-dominated scribes were observed on the bottom surface. Total internal reflection at the bottom surface allowed for more effective energy accumulation within the immediate vicinity. An ablation scribe could be formed on the bottom surface when sufficient energy had been accumulated. When the scanning speed was increased from 0.1 to 10 mm/s, energy deposition within the sample was reduced and thinner scribes were formed.

Laser irradiation at the upper section did not result in scribe formation on the bottom rough surface. Occasionally, with sufficient energy, the internal cracks propagated towards the bottom surface of the samples. At the slower scanning speeds of 0.1 and 1 mm/s, there was an excess of energy deposited, and thus cracks were observed on the bottom surface. The amount of energy deposition dictated the type of cracks that formed. At the 0.1 mm/s scanning speed, with more energy deposited, a long and curved crack was observed, while at the 1 mm/s scanning speed, with less energy deposited, short and faint cracks were observed. At the fastest scanning speed of 10 mm/s, there were no cracks observed on the bottom surface due to insufficient energy for internal crack propagation towards the bottom surface.

Figure 7c shows the optical images of the sidewall profiles of the successfully cleaved samples after one-third section scanning with different scanning speeds. The ultimate test for sample separation is the cleavage of the sample after laser scanning, and the surface scribes might not be the best indicator if cleavage is successful. As expected from the lack of visible modifications, mechanical cleavage was unsuccessful for samples scanned at the lower one-third section (i.e., Experiment L27S-50) for all scanning speeds. When the foci were located at the lower section, the energy loss due to linear absorption before arriving at the foci locations resulted in insufficient absorption at the lower section; therefore, the samples were not sufficiently weakened for cleavage.

Samples irradiated in the middle or upper one-third section (i.e., Experiments M27S-50 and U27S-50) could be cleaved when the scanning speed was either 0.1 mm/s or 1 mm/s. However, as shown in Figure 7c, samples irradiated at the fastest scanning speed of 10 mm/s could not be cleaved. This was attributed to the low energy deposition into the sample that could not effectively weaken the samples for cleavage. Even though the potential amount of energy absorption per pulse remained constant, the number of pulses deposited at a spot depended on the scanning speed. For example, a fast scanning speed would result in less pulse deposition at a spot and less energy would be absorbed.

At the slowest scanning speed of 0.1 mm/s, large amounts of energy were deposited onto the samples. This excess energy deposition formed large wave-like internal cracks with some inconsistent nonlinear modified areas for both upper and middle one-third section scanning experiments. These internal cracks prevented the propagation of the laser beam along the optical axis and the laser beam could not be properly focused at the intended foci positions. As a result, the laser intensity at these positions would be lower than expected; nonlinear absorption may not have occurred in these areas and consistent internal modifications could not be formed.

Increasing the scanning speed from 0.1 to 1 mm/s, with less energy deposited within the sample, the sidewall profile had more uniform internal modifications with shorter internal cracks. Since there was less excess energy to form the large internal wave-like cracks, the laser beam continued propagating along the optical axis with less obstructions. As a result of reduced scattering of light, the internal modifications by the laser were more consistent.

### 3.5. Mechanism of Wafer Cleavage with Energy Concentrated over Only One-Third Section of Thickness

The importance of a rough exit surface on enhancing total internal reflection within the sample could be explained by the scan of the middle one-third section in Experiment M27S-50. Due to the location of the foci, the amount of energy potentially absorbed by the sample would be less than that of Experiment U27S-50 (i.e., upper one-third section scans) but more than Experiment L27S-50 (i.e., lower one-third section scans). The sidewall profiles of the cleaved samples of Experiment M27S-50 consisted of two distinct types of modification regions. The first modification region was located near to the top surface, with large, dense and more consistent modifications. The occasional wave-like cracks were present above this modification region. The second modification region was located directly underneath the first region and was near to the bottom rough surface. This region had smaller modifications that were sparse and less consistent.

It was plausible that the first modification region was directly formed from the interaction with the incoming laser beam. As such, the energy deposition and accumulation at this region was sufficiently high to form the larger, denser and more consistent modifications. The excess energy deposition resulted in the formation of large wave-like cracks.

The second modification region might have been formed as a result of total internal reflection at the bottom rough surface. The total internal reflected light had a smaller amount of energy as some light would have been refracted out of the material. The incoming laser beam interacted with the totally internally reflected laser beam which then accumulated energy in this region. As a result of the lower laser energy accumulation in this region, smaller, sparser, and less consistent modifications were produced. The presence of these modifications illustrated that energy could indeed be accumulated near the bottom surface and explained the presence of the scribes observed on the bottom surface of the samples.

Increasing the scanning speed from 0.1 to 1 mm/s reduced the energy deposited within the sample. As a result, the sidewall profile had more uniform internal modifications in both regions, and shorter internal cracks.

In contrast, Experiment U27S-50 (i.e., upper one-third section scans) could not exploit the effects of total internal reflection as the foci were located far from the rough exit surface. With foci located in the upper one-third section, there was more energy potentially nonlinearly absorbed by the sample. Therefore, the excess energy absorbed resulted in more frequent formation of larger and longer internal cracks. These wave-like features hindered the focusing of the laser beam at the intended foci locations, resulting in less consistent nonlinear modifications observed on the sidewall. As a result, the sidewall profiles of cleaved samples in Experiment U27S-50 were less consistent and undesirable.

Since a uniformly roughened sidewall was ideal to enhance LED light extraction, Experiment M27S-50 (i.e., middle one-third section scans) was recommended as the amount of energy absorbed was optimal, producing more consistent nonlinear modifications with less cracks compared to Experiment U27S-50. A scanning speed of 1 mm/s was recommended as sample cleavage could be achieved with less energy. In addition, the sidewall profile had a greater uniformity of nonlinear modifications compared to that of the slower 0.1 mm/s scanning speed.

## 4. Conclusions

This investigation adopted the multi-foci technique to divide and allocate the pulse energy in the through thickness direction of the sapphire wafer for its singulation. Total internal reflection of laser beam exiting from an unpolished rough surface enhanced the nonlinear energy absorption for laser processing. This study has revealed that pulse energy division in the wafer thickness direction by splitting the laser energy over more foci with a smaller focus spot with closer proximity to each other is more effective for wafer separation than employing fewer foci with more energy and a larger focus spot but spaced further apart. To produce a cleavable sample, sufficiently high laser power density in the order of 10^12^ W/cm^2^ is critical to induce sufficient nonlinear absorption. Dividing and concentrating the pulse energy to only the middle one-third section of the wafer thickness over many foci with closer spatial proximity is sufficient, efficient and effective for wafer singulation. In addition, a rough sidewall profile desirable for improving light extraction of LEDs could be attained with this multi-foci laser irradiation of sapphire wafers.

## Figures and Tables

**Table 1:** Detailed optics combination for producing multi-foci and laser processing parameters.

	Shorter Foci Spacing over Entire Thickness	Larger Foci Spacing over Entire Thickness	Further Shorter Foci Spacing over One Third Thickness		
Experiment No.	F27	F9	L27	M27	U27
z value (μm) (Position of middle focus), (Total foci covering thickness section)	+210 Middle (M), Full section (F)	+210 Middle (M), Full section (F)	+360 Lower (L), 1/3 section	+210 Middle (M), 1/3 section	+70 Upper (U), 1/3 section
DOE (Foci No),	27	9	27	27	27
Focusing optic EFL (mm)	7.5	12	4.38 (OB40×)	4.38 (OB40×)	4.38 (OB40×)
Foci spot diameter (μ **m)**	2.20	3.52	1.29	1.29	1.29
Ave foci spacing (μm)	15.4	43.6	5.2	5.2	5.2
Total foci spacing (μm)	399.5	348.9	136.3	136.3	136.3
Samples tested (S or D)	Single and double polished (S&D)	Single polished (S)	Single polished (S)	Single polished (S)	Single polished (S)
Laser power (W)	1.13	1.13	0.80	0.80	0.80
Pulse repetition rates (kHz)	50, 500, 5120	50	50	50	50
Pulse energy per focus (μJ)	0.837, 0.0837, 0.00817	2.511	0.593	0.593	0.593
Pulse energy fluence per focus (J/cm^2^)	594.8, 59.48, 5.809	232.355	1224.816	1224.816	1224.816
Power per pulse per focus (W)	8.126 × 10^4^, 8.126 × 10^3^, 0.794 × 10^3^	2.438 × 10^5^	5.753 × 10^4^	5.753 × 10^4^	5.753 × 10^4^
Power density per pulse per focus (W/cm^2^)	2.14 × 10^12^, 2.14 × 10^11^, 2.09 × 10^9^	2.506 × 10^12^	4.404 × 10^12^	4.404 × 10^12^	4.404 × 10^12^

(1) “F”, “L”, “M” and “U” indicate the scanning position, meaning that the sample was scanned across the full thickness, lower, middle or upper one-third sample section, respectively; (2) the numbers “9” and “27” indicate the number of foci of the laser beam; (3) “S” or “D”, respectively indicate a single-side polished or double-side polished sample; (4) the number after “S” or “D” indicates the pulse repetition rate in kHz; (5) for example, Experiment F27D-50 indicates that the experiment was carried out on a double-side polished sample with 27 foci laser beam distributed across the entire section with a pulse repetition rate of 50 kHz.

## Data Availability

Not applicable.
